# Challenging cases discussed by experts: retinal vasculitis following coinfection with HIV and syphilis

**DOI:** 10.1007/s12348-011-0022-1

**Published:** 2011-04-22

**Authors:** Thomas Albini, Janet L. Davis, Claudio D. Tuda

**Affiliations:** 1 Bascom Palmer Eye Institute, 900 NW 17th St, Miami, FL 33136 USA; 2 4300 Alton Road #203, Miami, FL USA

**Keywords:** Syphillis, HIV, Retina, Vasculitis

## Abstract

A patient with HIV and syphilis presents with bilateral retinal vasculitis and recurrent vitreous hemorrhage. Diagnostic and treatment strategies are discussed.

## Case report

A 42-year-old human immunodeficiency virus (HIV)-infected male on highly active antiretroviral therapy (HAART) was referred for new floaters in the left eye. He was diagnosed with HIV 6 years prior when he presented with disseminated Kaposi’s sarcoma with lymph node involvement. A year later, he was diagnosed with human herpes virus 8 (HHV-8)-associated primary effusion lymphoma and successfully treated with 6 months of oral valganciclovir. He had not sustained any opportunistic infections. The most recent CD4 T-cell count was 720 cells/mm^3^, and the HIV viral load was undetectable.

Past medical history included a nonpruritic rash of the trunk 18 months prior to presentation associated with an RPR of 1:128. Positron emission tomography–computed tomography showed diffuse lymphadenopathy. Subsequent lymph node biopsy revealed reactive lymphadenitis, thought to be secondary to syphilis. He was treated twice with three weekly intramuscular injections of 2.4 million units of benzathine penicillin (PCN) over the subsequent year.

**Fig. 1:**
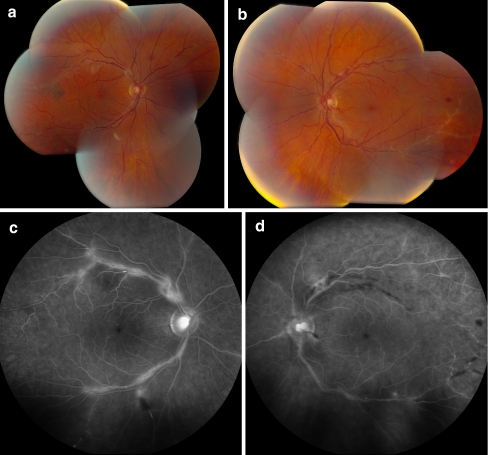
A 42-year-old HIV-positive male with recent onset floaters. Segmental sheathing and beading of arterioles and venules extend most prominently along the superotemporal arcades in the right eye (**a**). In the left eye, sheathing of the arteries and veins is most prominent along the inferotemporal arcade with peripapillary and intraretinal hemorrhage (**b**). Fluorescein angiography demonstrates normal arterial filling. The venous filling is segmental and shows areas of perivenous hypofluorescence in the right eye (**c**) and left eye (**d**)

At presentation, visual acuity was best corrected to 20/25 OD and 20/30 OS. Intraocular pressures were 31 mmHg OD and 15 mmHg OS. There was no afferent pupillary defect. No keratic precipitates or posterior synechiae were present in either eye. The anterior chamber of the right eye exhibited trace anterior chamber cell and the left eye, 1+ anterior chamber cell. There was approximately 1+ anterior vitreous cell in the right eye and 3+ anterior vitreous cell in the left eye. Dilated funduscopy of the right eye revealed a healthy-appearing optic nerve with a cup-to-disk ratio of 0.3. Segmental sheathing of arterioles and venules extended most prominently along the superotemporal arcades (Fig. 1a). Cotton wool spots were found throughout the posterior pole. Examination of the left eye disclosed similar findings (Fig. 1b). Fluorescein angiography demonstrated normal arterial filling. The venous filling in both eyes was segmental and showed areas of perivenous hypofluorescence (Fig. 1c and d).

An evaluation for causes of retinal vasculitis revealed an RPR of 1:8 and a positive FTA-ABS. Cytomegalovirus (CMV) IgG antibody, varicella zoster virus (VZV) IgG, herpes simplex virus (HSV)-1 IgG, HSV-2 IgG, and Epstein–Barr virus IgG antibodies were all present. Quantitative PCR for Epstein Barr virus and CMV measured 12,456 copies per milliliter and less than 200 copies per milliliter, respectively. Cardiolipin antibody was not present. ANCA screen was negative. ANA was positive with a titer of 1:80 in a speckled pattern and a titer of 1:40 in a nucleolar pattern. Protein C and S activity were both normal. The HLA B51 haplotype was present. A brain MRI and MRA were both unremarkable.

Intravenous PCN sodium (24 million units daily) was administered for 14 days. Over the subsequent 6 months, the RPR titer decreased to 1:4. One month following presentation, he developed a vitreous hemorrhage in his left eye associated with retinal neovascularization and vasculitis. Due to the progression of the eye disease, a lumbar puncture was obtained which showed a clear cerebrospinal fluid (CSF) without pleocytosis; CSF analysis including VDRL, cytology, flow cytometry, and PCR for VZV and HSV was negative.

Six weeks after presentation, a diagnostic vitrectomy was performed in the left eye. PCR for *Mycobacterium tuberculosis*, HSV, HHV-8, toxoplasmosis, VZV, Epstein–Barr virus, cytomegalovirus, and *Treponema pallidum* were all negative. Flow cytometry disclosed only 43 viable cells, without any definable pattern.

**Fig. 2:**
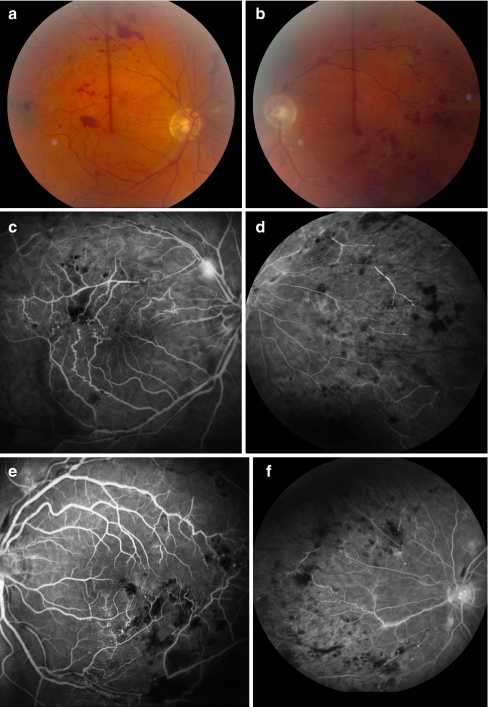
Seven weeks later, the degree of vascular sheathing, beading, and tortuosity has decreased, while intraretinal hemorrhage and exudate have increased in both eyes (**a** and **b**). Early-phase fluorescein angiography, right eye (**c**) and left eye (**e**), shows a blocking defect by retinal hemorrhage, vascular occlusion, and aneurysmal changes. Early frames of the nasal retina of the right eye (**d**) and left eye (**f**) show vascular leakage and global areas of non-perfusion

Seven weeks subsequent to presentation, extensive areas of vascular occlusion were noted on angiography (Fig. 2). Twelve weeks after presentation, visual acuity remained 20/20 OU. Fluorescein angiography disclosed areas of persistent occlusive vasculitis, advancing areas of retinal nonperfusion, and neovascularization. Both eyes were treated with panretinal laser photocoagulation. Over the subsequent 4 months, the patient experienced three vitreous hemorrhages in the right eye and two in the left, all of which resolved with observation.

Further panretinal photocoagulation will be performed in both eyes, and the therapeutic options considered include observation, systemic immunosuppression, immunomodulation with interferon, or further treatment for neurosyphilis with antibiotics and with or without systemic steroids.

Questions:

1. Is syphilis the underlying cause of this patient’s vasculitis?
2. Is the HLA-B51 haplotype relevant to his disease?
3. Are any other diagnostic studies warranted?
4. Which treatments other than photocoagulation are likely to be helpful?

## Expert comments

Dr. James P. Dunn, Baltimore, MA, USA

1. Is syphilis the underlying cause of this patient’s vasculitis?
  Syphilis remains the most likely diagnosis. The failure to respond to intramuscular benzathine penicillin reinforces the premise that ocular syphilis in patients with HIV/AIDS should always be treated as neurosyphilis, with high-dose intravenous penicillin (12–24 million units per day in divided dosages for 10–14 days).
  I would like more information about the patient. For example, is there any clinical condition to support a possible diagnosis of Behçet’s disease, such as oral or cutaneous ulcerations? What medications is the patient taking? Is there a current history of cigarette smoking?
2. Is the HLA-B51 haplotype relevant to his disease?
  The HLA-B51 haplotype is unlikely to be relevant. Patients with Adamantiades–Behçet’s disease are more likely to carry this gene than other patients with uveitis, but this disease is diagnosed clinically. In the absence of other symptoms (oral ulcers, genital ulcers, cutaneous hypersensitivity–not the truncal rash described in this patient–, etc.), which this patient does not appear to have, I would not consider the HLA-B51 test to be of clinical significance in this case. It remains unclear what the predictive positive value of this test is in patients who have only ocular findings consistent with Behçet’s disease.
  On the other hand, HLA-B51 is found more frequently in patients with Behçet’s disease who have ocular involvement. However, if the B51 test is accorded any weight, then anti-TNF therapy such as infliximab, which may be of particular benefit in patients with Behçet’s disease, could be considered.
3. Are any other diagnostic studies warranted?
  I would get a QuantiFERON TB gold test, because tuberculosis could still be in the differential diagnosis (although unlikely). HLA-B51 subtypes could be considered, such as B5101, which may be more suggestive of Behçet’s disease.
4. Which treatments other than photocoagulation are likely to be helpful?
  I would add oral corticosteroids (prednisone) at a dose of 60 mg/day and would consider intravitreal therapy, including preservative-free triamcinolone acetonide, 4 mg, or the intravitreal dexamethasone implant, 750 μg, if oral therapy appears effective but is not tolerated. In addition, intravitreal anti-VEGF therapy (bevacizumab or ranibizumab) can also be considered for the neovascularization, although there is a risk of worsening the ischemic component.

Dr. Ramana Moorthy, Indianapolis, IN, USA

1. Is syphilis the underlying cause of this patient’s vasculitis?
  Based on epidemiologic and clinical evidence, syphilis is most likely the underlying cause of this patient’s vasculitis. The incidence of primary and secondary syphilis is increasing and is particularly prevalent in men having sex with men, 50–60% of whom are already known to be HIV infected [1]. Manifestations of syphilis are protean but can be atypical in HIV-positive patients. Occlusive retinal vasculitis can occur [2]. The initial serologic and clinical evidence suggests inadequately treated post-primary syphilis in this patient despite immune reconstitution (CD4 = 720 cells/mm^3^ [3]). Retreatment with intravenous penicillin reduced serum RPR titers, but they remained detectable (1:4). The vasculitis progressed, and retinal neovascularization developed indicating inadequate response to treatment. CSF VDRL was negative. Vitreous PCR for *Treponema pallidum* was negative.
  Other infectious causes of occlusive vasculitis including tuberculosis, Lyme disease, necrotizing herpetic retinitis were ruled out serologically or by PCR of intraocular fluids. Non-infectious systemic causes of occlusive retinal vasculitis include Behçet’s disease (BD), systemic lupus erythematosus, Wegener’s granulomatosis, and polyarteritis nodosa. All except BD were ruled out by serology.
2. Is the HLA-B51 haplotype relevant to the patient’s disease?
  Although syphilitic retinal vasculitis was suspected and treated, the disease progressed raising concern about non-syphilitic causes. Workup revealed the presence of the HLA-B51 haplotype, raising concern about BD. It is not a laboratory parameter that I routinely obtain in patients with retinal vasculitis. The HLA-B51 allele has a strong association with Behçet’s disease [3]. In the absence of these non-ocular clinical manifestations, however, the mere presence of the HLA-B51 haplotype may not be enough to diagnose BD. To date, here have been ten reported cases of BD in HIV-infected patients [4]. Many of the posterior segment ocular opportunistic infections in HIV-simulated findings of ocular BD [4]. Immunomodulatory therapy was safely utilized in all of these cases in combination with HAART (to maintain immune reconstitution and undetectable viral loads) with the majority of patients recovering from their BD [4].
3. Are any other diagnostic studies warranted?
  A thorough review of systems looking at manifestations of BD or systemic vasculitis should be performed. I would recommend a repeat HIV viral load, CD4 count, and QuantiFERON TB Gold test. Although there are no other specific diagnostic studies for BD, cutaneous pathergy testing may be useful.
4. Which other treatments other than photocoagulation are likely to be helpful?
  Since the RPR titer is still low–positive at 1:4, I would recommend retreatment of the patient based on neurosyphilis protocol of 14 days of high doses of intravenous penicillin therapy to ensure that the RPR serology becomes negative. I would recommend intravitreal anti-VEGF therapy (bevacizumab or ranibizumab) and additional panretinal photocoagulation of untreated areas of angiographic retinal ischemia in each eye for persistent retinal neovascularization. If this approach fails to control the retinal vasculitis, then BD or other non-infectious causes of retinal vasculitis must be considered and immunomodulatory therapy instituted with caution. HAART must be continued. Prednisone, cyclosporine, and colchicine have all been used with HAART in HIV-positive patients with BD [4]. Although TNF-α inhibitors (infliximab) are excellent treatment for ocular BD, the safety of these agents in patients with HIV infection is unknown. The immunomodulatory and anti-viral effects of interferon alpha may be uniquely beneficial in patients with HIV and BD, but little data exist to recommend such therapy.

## Section editor’s note

Albini and colleagues present an interesting case of a middle-aged man coinfected with HIV and *Treponema pallidum*, who presented with severe bilateral retinal vasculitis, progressing despite treatment with intravenous penicillin.

Indeed, syphilitic uveitis is reemerging, particularly in association with HIV infection [2, 5, 6]. According to both invited experts, Drs. Dunn and Moorthy, syphilis is likely associated with such retinal vaso-occlusive process in this HIV-infected patient, even despite apparently appropriate therapy for neurosyphilis. Of note, the RPR titer decreased, but was still measurable (low titer of 1:4) after treatment, what might indicate that the patient was not definitely cured. Even though high-dose intravenous crystalline penicillin is regarded as the gold-standard therapy for neurosyphilis, there are concerns about resistance and limited antibiotic penetration in the CNS [7], as well as inside the eye [8].

Comprehensive investigations revealed positive testing for HLA-B51, raising the suspicion of a Behçet’s disease background. The experts are also unanimous that the presence of HLA-B51 in this patient may be coincidental, especially in the absence of other signs and symptoms of Behçet’s disease. In case this positive HLA-B51 result were to be valued for diagnosing the patient with Behçet’s disease, Drs. Dunn and Moorthy suggest that appropriate immunomodulatory therapy would have to be considered, as has been anecdotally reported in patients with both HIV and Behçet’s disease [4]. The positive ANA may also raise some suspicion of lupus erythematosus

Regarding additional testing, an interferon-gamma release assay would be helpful to exclude tuberculosis, according to both invited experts. It is also important to note that a negative PCR for infectious agents does not definitely exclude them [9].

In addition to the treatment for neurosyphilis with intravenous crystalline penicillin, Dr. Dunn also suggests a trial with oral prednisone, 60 mg daily. Dr. Moorthy would consider immunomodulatory therapy, if the patient does not respond to retreatment for neurosyphilis with penicillin. Both experts also suggest intravitreal anti-VEGF therapy, to control the retinal neovascularization, besides laser photocoagulation. This should be done with caution, due to the risk of aggravation of ischemia [10].

We thank Dr. Albini and colleagues for submitting this interesting case, and the invited experts for their precious comments.
